# Care practices and short-term clinical outcomes of very low birth weight infants in Yangtze River Delta in China

**DOI:** 10.1186/s12887-022-03749-6

**Published:** 2022-11-23

**Authors:** Tianchan Lyu, Yibo Tao, Wei Hua, Liling Li, Yunfei Tang, Yumei Jin, Yan Wang, Yuelan Ma, Futing Ji, Yalan Dou, Yun Cao, Xiao-jing Hu

**Affiliations:** 1 Children’s Hospital of Fudan University, Shanghai, China; 2 Wuxi Children’s Hospital, Wuxi, Jiangsu China; 3 Ningbo Women and Children’s Hospital, Ningbo, Zhejiang China; 4 Anhui Provincial Children’s Hospital, Hefei, Anhui China; 5 Suzhou Municipal Hospital, Suzhou, Jiangsu China

**Keywords:** Very low birth weight infant, Extremely low birth weight infant, Care, Outcome

## Abstract

**Background:**

Intensive care is of great significance for very low birth weight infants (VLBWI). The Yangtze River Delta is the most ecomonically developed area in China. However, there are few data on the care practices and survival of VLBWI in this region.

**Objectives:**

To investigate the prevalence, care practices and motality of VLBWI in Yangtze River Delta in China.

**Methods:**

A multi-center retrospective investigation study was conducted at five tertiary hospitals within the Yangtze River Delta in China from January to December 2017. Clinical data included the general characteristics of the infants and the mothers, clinical prognosis, care practices in NICUs was collected by trained research members.

**Results:**

During the study period, 1059 VLBWIs were included. Infants with birth weight < 750 g, 750-1000 g, 1000-1250 g and 1250-1500 g accounted for 2.3, 14.9, 34.8 and 47.8%, respectively. Premature rupture of membranes (17.8%) was the main cause of premature delivery. The catheterization rates of umbilical vein catheterization (UVC) and peripherally inserted central catheter (PICC) were 25.0 and 64.4%, respectively. The duration of parenteral nutrition was 27.0 ± 19.5 d, the meantime of feeding tube indwelling was 36.2 ± 24.2 d. The corrected gestational age of the infants who reached full oral feeding was 35.8 ± 2.7 weeks. The breast feeding rate in the investigated infants was 61.9%. The mortality rate of preterm infants was 3.4%. The incidence of main complications BPD, PDA, ROP, NEC and sepsis were 24.9, 29.9, 21.7, 9.4 and 13.3% respectively.

**Conclusions:**

Maternal and infant care practices need to be improved in the very preterm births. This study provides a baseline for the improvement in the further study.

## Background

Very low birth weight infants (VLBWI) are defined as neonates with birth weight of less than 1500 g. VLBWI accounted for 20% of total hospitalized infants in the neonatal intensive care units (NICU), while extremely low birth weight infants (ELBWI) with a birth weight of less than 1000 g accounted for 15.1% [1]. According to previous study, high mortality of VLBWI was observed [2]. In China, VLBWI has been reported to account for 1/3 of the deaths in NICU, [3] with the total VLBWI-related mortality reaching 23.9% (705/2956) in 2018 [1]. VLBWI has suffered from multiple neonatal complications, such as sepsis, bronchopulmonary dysplasia (BPD), intraventricular hemorrhage (IVH), periventricular leukomalacia (PVL), necrotizing enterocolitis (NEC), and retinopathy of prematurity (ROP), which can aggravate the infant condition, increase medical costs, and prolong hospital stay [4, 5]. Poor neurodevelopmental prognosis has been reported among VLBWI [6].

With the rapid development of perinatal medicine and neonatal intensive care in China, more VLBWI have been admitted and treated in Chinese NICUs. Several epidemiological studies have been conducted aiming to describe the prevalance and prognosis of VLBWI in NICU. These data were widely used to assist clinical decision-making, evaluate the quality of neonatal treatment, and carry out quality improvement of NICU [1, 3, 7]. The Yangtze River Delta is the most econonmically developed area in China, which is a representative area. In this region, the incidence of VLBW is higher, and the quality of care is relatively good. However, there are few data on the care practices and survival of VLBWI in this region. The objectives of our study were to analyze care practices, motality and morbidity of VLBWIs in the Yangtze River Delta, so as to provide reference data for clinical prognosis of preterm infants, and baseline data for clinical treatment and care practices of VLBWIs.

## Methods

### Study design, population and setting

A multi-center cohort observational study was conducted in NICUs at 5 tertiary hospitals within the Yangtze River Delta in China. The Yangtze River Delta region incorporate Shanghai District, Jiangsu Province and Zhejiang Province. It is one of the most densely populated regions on earth, with over 150 million registered residents living in more than twenty developed cities including Nanjing, Hangzhou, Suzhou, Ningbo, Wuxi, and Changzhou. All the participating hospitals were tertiary neonatal care centers. The data came from the care database of VLBWIs in the Yangtze River Delta. All preterm infants with a weight of less than 1500 g who were younger than seven days at admission were included and the data from admission to discharge or death were all in the database. The data of all stillbirths and infants who were not discharged or died in these hospitals were not in the database. The data of infants discharged from January 1, 2017, to December 31, 2017 were analyzed in this study. Infants’ guardian signed informed consent. The study was approved by Ethics committee of Children’s Hospital of Fudan University ([2018]-211).

### Data collection

The collected data included the general characteristics of the infants and the mother, clinical prognosis, conditions of nursing related care in NICU. Each unit designated a special data collector (senior neonatal nurse) who was responsible for data collection. All data collectors were trained before the study. The data of each unit was uploaded to the special database established for the VLBWI project in the Yangtze River Delta. The research group located at Children’s Hospital of Fudan University (CHFU) was responsible for checking the integrity and quality of the data. In addition, members of different research centers traveled to other centers for data audit. All data for this study were collected from the electronic medical record system of each hospital.

### Data definition

The important data involved in the study were defined as follows: (1) gestational age: defined as the gestational age evaluated by early pregnancy ultrasound, obstetric examination, and obstetric history. If the difference between obstetric gestational age and neonatal gestational age assessment in NICU was more than 2 weeks, the neonatal simple gestational age assessment form was used for the assessment. The New Ballard score was used to evaluate the gestational age of preterm infants less than 28 weeks [8]. (2) Use of prenatal corticosteroids: the mother received glucocorticoids before delivery. (3) BPD: infants needed oxygen at 36 weeks of corrected gestational age or at discharge [9]. (4) Intraventricular hemorrhage (IVH): according to the results of B-ultrasound and according to the Papile criteria, grade III IVH was defined as intraventricular hemorrhage with ventricular enlargement, while grade IV IVH as intraventricular hemorrhage with periventricular hemorrhage [10]. (5) Necrotising enterocolitis (NEC): diagnosis and staging according to Bell [11]. (6) Retinopathy of prematurity (ROP): diagnosis and staging were performed according to ROP International Diagnostic and Staging criteria [11]. (7) Sepsis: sepsis with positive blood culture or clinical sepsis with negative blood culture but accompanied by clinical symptoms or signs [12]. (8) Total oral feeding: total enteral nutrition without tube feeding for at least 48 hours [13].(9) Hypothermia: defined as anytime axillary temperature less than 36.5 °C during hospitalization [14]. (10) Hyperthermia: defined as anytime axillary temperature more than 37.5 °C during hospitalization [14].

### Statistical analysis

SPSS Version 22.0 software was used for statistical analysis of all data. For the objective of this study, the status of care measures, mortality & morbidity of neonatal outcome were described and summarized among VLBWIs. Continuous variables with normal distribution were expressed as means ± Standard deviation while those with highly-skewed distribution were represented by the median (p25-p75). Categorical variables were expressed as number of case (percentage). The denominator for percentages was the total number of infants with no missing data for each variable analyzed. Missing data were not imputed. For formal statistical analyses, we examined categorical variables using χ^2^ tests and continuous data using analysis of variance (ANOVA). A *p*-value < 0.05 was considered to be statistically significant with two-tail test.

## Results

A total of 1059 VLBWIs with an average gestational age of 29.8 ± 2.3 weeks and an average birth weight of 1216.6 ± 198.0 g were included in the study. Infants with birth weight < 750 g, 750-1000 g, 1000-1250 g and 1250-1500 g accounted for 2.3% (25 infants), 14.9% (158 infants), 34.8% (369 infants) and 47.8% (507 infants), respectively. Among them, 183 infants (17.2%) were ELBW, while 126 infants (11.9%) were infants with multiple pregnancies. Of total mothers of 1059 infants, 86.0% (911 mothers) had a regular prenatal examination, 21.1% (223 mothers) received prenatal glucocorticoids. Among the causes of preterm delivery, preterm rupture of membranes, fetal distress, placental abruption, and placenta previa accounted for 17.9, 4.4, 3.1, and 14.5%, respectively. The proportion of Apgar score in 1 min less than 3 reduced as the increase of birth weight, see Tables 1 and 2.

**Table 1:** Maternal characteristics of the study population

Characteristics	≤750 g (***n*** = 25)	751-1000 g(***n*** = 158)	1001-1250 g(***n*** = 369)	1251-1500 g(***n*** = 507)	Total(***n*** = 1059)	F/χ^**2**^	***P***	***P*** value for Trend
Maternal Age,mean(SD),y	30.3 ± 4.6	31.7 ± 5.6	30.5 ± 5.2	30.2 ± 5.4	30.6 ± 5.4	2.911	0.034	0.023
Standard prenatal care(n，%)	24(96.0%)	145(91.8%)	315(85.4%)	427(84.2%)	911(86.0%)	7.916	0.048	0.025
Primary diabetes mellitus(n，%)	0(0.0%)	0(0.0%)	4(1.1%)	7(1.4%)	11(1.0%)	2.505	0.474	0.142
Gestational diabetes mellitus(n，%)	6(24.0%)	20(12.7%)	48(13.0%)	67(13.2%)	141(13.3%)	2.567	0.463	0.580
Essential hypertension(n，%)	0(0.0%)	1(0.6%)	12(3.3%)	6(1.2%)	19(1.8%)	7.190	0.066	0.913
Pregnancy induced hypertension(n，%)	2(8.0%)	24(15.2%)	53(14.4%)	76(15.0%)	155(14.6%)	0.993	0.803	0.654
Prenatal glucocorticoid use(n，%)	13(52.0%)	74(46.8%)	199(53.9%)	223(44.0%)	223(21.1%)	8.697	0.034	0.080
Premature rupture of membranes(n，%)	0(0.0%)	16(10.1%)	57(15.5%)	116(22.9%)	189(17.9%)	22.062	0.000	0.000
Fetal distress(n，%)	2(8.0%)	3(1.9%)	17(4.6%)	25(4.9%)	47(4.4%)	3.465	0.325	0.382
Multiple pregnancy(n，%)	10(40.0%)	20(12.7%)	38(10.3%)	58(11.4%)	126(11.9%)	19.924	< 0.001	0.037
Placental abruption(n，%)	0(0.0%)	10(6.3%)	6(1.6%)	17(3.4%)	33(3.1%)	9.015	0.029	0.606
Placenta previa(n，%)	4(16.0%)	32(20.3%)	55(14.9%)	63(12.4%)	154(14.5%)	6.055	0.109	0.025

**Table 2:** Neonatal characteristics of the study population

Characteristics	≤750 g (***n*** = 25)	751-1000 g(***n*** = 158)	1001-1250 g(***n*** = 369)	1251-1500 g(***n*** = 507)	Total(***n*** = 1059)	F/χ^**2**^	***P***	***P*** value for trend
Male(n，%)	13(52.0%)	74(46.8%)	214(57.9%)	282(55.6%)	583(55.1%)	5.791	0.122	0.172
Gestational Age (weeks)	27.2 ± 2.6	27.9 ± 2.0	29.5 ± 1.9	30.8 ± 2.0	29.8 ± 2.3	108.027	0.000	0.000
Birth Weight (g)	686.0 ± 45.3	918.6 ± 69.5	1151.4 ± 71.4	1383.1 ± 69.7	1216.6 ± 198.0	2704.138	0.000	0.000
1 min Apgar score < 3(n，%)	2(8.0%)	9(5.7%)	14(3.8%)	0(0.0%)	25(2.3%)	17.296	0.001	0.000
5 min Apgar < 3(n，%)	0(0.0%)	2(1.3%)	3(0.8%)	1(0.2%)	6(0.6)	4.441	0.218	0.716
Cesarean section(n，%)	12(23.1%)	90(56.9%)	176(47.7%)	203(40.1%)	481(45.4%)	15.250	0.002	0.000

### Respiratory care practices

Rate of pulmonary surfactant use increased with decreased birth weight, from 64% at < 1000 g to 43% at 1251-1500 g. The top three utilization of respiratory support modes were CPAP (59.8%), conventional mechanical ventilation (CMV, 31.9%), and nasal tube oxygen inhalation (39.7%). Of total VLBWI, 688 (65.0%) infants, 536 (50.6%) infants, 207 (19.6%) infants were treated with caffeine, PS and aerosol inhalation respectively, see Table 3.

**Table 3:** Respiratory care practices of the study population

Respiratory therapy	≤750 g (***n*** = 25)	751-1000 g(***n*** = 158)	1001-1250 g(***n*** = 369)	1251-1500 g(***n*** = 507)	Total(***n*** = 1059)	χ^**2**^	***P***	***P*** value for trend
HFV(n，%)	13(52.0%)	46(29.1%)	68(18.4%)	42(8.0%)	169(15.7%)	49.734	0.000	0.000
CMV(n，%)	12(48.0%)	81(51.3%)	128(34.7%)	117(23.0%)	338(31.9%)	49.734	0.000	0.000
NHFO(n，%)	0(0.0)	6(3.8%)	4(1.1%)	5(1.0%)	15(1.4%)	7.738	0.052	0.129
BIPAP(n，%)	8(32.0%)	57(36.1%)	98(26.6%)	60(12.0%)	223(21.1%)	55.900	0.000	0.000
CPAP(n，%)	9(36.0%)	95(60.1%)	241(65.3%)	288(57.0%)	633(59.8%)	12.450	0.006	0.835
NIPPV(n，%)	4(16.0%)	30(19.0%)	75(20.3%)	58(11.0%)	167(15.8%)	14.154	0.003	0.020
HHFNC(n，%)	7(28.0%)	62(39.2%)	122(33.1%)	118(23.0%)	309(29.2%)	19.004	0.000	0.004
Nasal catheter(n，%)	8(32.0%)	61(38.6%)	166(45.0%)	185(36.0%)	420(39.7%)	9.191	0.066	0.889
Nose / face mask(n，%)	1(4.0%)	20(12.7%)	57(15.5%)	75(15.0%)	173(16.3%)	6.700	0.082	0.002
PS(n，%)	16(64.0%)	102(64.6%)	200(54.2%)	218(43.0%)	536(50.6%)	27.744	0.000	0.000
Caffeine(n，%)	19(76.0%)	121(76.6%)	256(69.4%)	292(58.0%)	688(65.0%)	25.966	0.000	0.000
Aerosol Inhalation (n，%)	7(28.0%)	54(34.8%)	82(22.2%)	64(13.0%)	207(19.6%)	40.702	0.000	0.000

*CMV=*Conventional Mechanical Ventilation, *HFV=*High Frequency Ventilation, *NHFO=*Noninvasive High Frequency Oscillation Ventilation, *BIPAP=*Biphasic Intermittent Positive Airway Pressure, *CPAP=*Continuous Positive Airway Pressure, *NIPPV=*Noninvasive Positive Pressure Ventilation, *HHFNC=*Humidified High Flow Nasal Cannula, *PS=*Pulmonary Surfactant

### Vascular access choices

Umbilical vein catheterization (UVC, 25.0%) and Peripheral central venous catheterization (PICC, 64.4%) were the most commonly used for vascular accesses in VLBWI. Among VLBWIs undergoing UVC, the mean indwelling time was 6.7 ± 4.8 days. The mean catheterization time of VLBWIs with PICC was 23.7 ± 16.2 days. See Table 4.

**Table 4:** Vascular access of the study population

Treatment	≤750 g (***n*** = 25)	751-1000 g(***n*** = 158)	1001-1250 g(***n*** = 369)	1251-1500 g(***n*** = 507)	Total(***n*** = 1059)	F/χ^**2**^	***P***	***P*** value for trend
UAC(n，%)	6(24.0%)	21(13.3%)	43(11.7%)	18(3.6%)	88(8.3%)	33.710	0.000	0.000
UVC(n，%)	12(48.0%)	59(37.3%)	122(33.1%)	72(14.2%)	265(25.0%)	64.173	0.000	0.000
PICC(n，%)	11(44.0%)	106(67.1%)	243(65.9%)	322(63.5%)	682(64.4%)	5.551	0.136	0.071
CVC(n，%)	1(4.0%)	0(0.0%)	1(0.3%)	6(1.2%)	8(0.8%)	7.107	0.069	0.342

*UAC=*Umbilical Atery Catheter, *UVC=*Umbilical Vein Catheter, *PICC=*Peripherally Inserted Central Catheter, *CVC=*Central Venous Catheterization

### Nutritional care practices

The duration of parenteral nutrition was 27.0 ± 19.5 days and the average duration of feeding tube indwelling was 36. 2 ± 24.2 days. The corrected gestational age of the VLBWI who reached full oral feeding was 35.8 ± 2.7 weeks in this study. The exclusive breast feeding rate was 61.9% among VLBWI (Table 5).

**Table 5:** Nutritional care practices of the study population

Treatment	≤750 g (***n*** = 25)	751-1000 g(***n*** = 158)	1001-1250 g(***n*** = 369)	1251-1500 g(***n*** = 507)	Total(***n*** = 1059)	F/χ^**2**^	***P***	***P*** value for trend
Days of enteral nutrition (d)	21.8 ± 24.8	30.9 ± 24.9	26.3 ± 18.7	22.4 ± 16.2	27.0 ± 19.5	7.560	0.000	0.447
Indwelling time of feeding tube (d)	42.1 ± 37.7	43.4 ± 32.2	38.2 ± 23.8	27.4 ± 17.9	36.2 ± 24.2	25.267	0.000	0.000
PMA at full oral feeding (week)	36.8 ± 3.5	37.3 ± 3.6	36.2 ± 2.5	35.2 ± 2.4	35.8 ± 2.7	19.294	0.000	0.000
Exclusive breast milk(n，%)	15(60.0%)	102(64.6%)	235(63.7%)	303(59.8%)	655(61.9%)	1.989	0.575	0.281
Breastfeeding by his/her own milk(n，%)	14(56.0%)	95(60.1%)	222(60.2%)	286(56.4%)	617(58.3%)	1.542	0.673	0.397

### Monitor care practices

Hypothermia occurred in 11.7% of study population during hospitalization. The incidence of Hypothermia decreased significantly with the increase of birth weight: 28% at infants < 750 g, 19% at 750-1000 g, 13.3% at 1250-1500 g and 7.5% at 1250-1500 g. The proportion of VLBWI undergoing EEG monitoring, continuous glucose monitoring and percutaneous partial pressure of oxygen monitoring was 16.8, 15.8 and 20.9% respectively (Table 6).

**Table 6:** Monitor care practices and neonatal outcome of the study population

Variables	≤750 g (***n*** = 25)	751-1000 g(***n*** = 158)	1001-1250 g(***n*** = 369)	1251-1500 g(***n*** = 507)	Total (***n*** = 1059)	F/χ^**2**^	***P***	***P*** value for Trend
Hypothermia(n，%)	7(28.0%)	30(19.0%)	49(13.3%)	38(7.5%)	124(11.7%)	24.103	0.00	0.00
Hyperthermia(n，%)	6(24.0%)	39(24.7%)	96(26.0%)	118(23.3%)	259(24.5%)	1.936	0.93	0.62
EEG monitoring(n，%)	5(20.0%)	38(24.1%)	63(17.1%)	72(14.2%)	178(16.8%)	8.592	0.04	0.01
Continuous glucose monitoring(n，%)	1(4.0%)	17(10.8%)	51(13.8%)	98(19.3%)	167(15.8%)	11.485	0.01	0.00
Intestinal or cerebral oxygen monitoring(n，%)	1(4.0%)	4(2.5%)	10(2.7%)	10(2.0%)	25(2.4%)	0.839	0.84	0.44
Percutaneous partial pressure of oxygen monitoring(n，%)	3(12.0%)	37(23.4%)	74(20.1%)	107(21.1%)	221(20.9%)	1.978	0.58	0.84

### Neonatal outcomes

The smaller of the birth weight, there was a greater risk of mortality due to prematurity. The mortality rate of VLBWI was 32% at < 750 g, 6.3% at 750-1000 g, 3% at 1000-1250 g and 1.4% at 1250-1500 g. There was significantly reduction of incidence of Sepsis with the increase of birth weight: 28% at < 750 g, 19.6% at 750-1000 g, 15.2% at 1000-1250 g and 9.3% at 1250-1500 g. The incidences of BPD, PDA, ROP and NEC were 24.9, 29.9, 21.7 and 9.4%, respectively. There were significant differences in the incidence of BPD, ROP and NEC among different birth weight groups (Table 7).

**Table 7:** Neonatal outcome of the study population

Variables	≤750 g (***n*** = 25)	751-1000 g(***n*** = 158)	1001-1250 g(***n*** = 369)	1251-1500 g(***n*** = 507)	Total (***n*** = 1059)	F/χ^**2**^	***P***	***P*** value for Trend
Death	8(32.0%)	10(6.3%)	11(3.0%)	7(1.4%)	36(3.4%)	97.351	0.00	0.00
BPD(n，%)	4(16.0%)	67(42.4%)	117(31.7%)	76(15.0%)	264(24.9%)	62.441	0.00	0.00
NEC(n，%)	3(12.0%)	14(8.9%)	40(10.8%)	42(8.3%)	99(9.4%)	1.898	0.59	0.43
ROP(n，%)	1(4.0%)	60(38.0%)	93(25.2%)	76(15.0%)	230(21.7%)	46.241	0.00	0.00
PDA(n，%)	7(28.0%)	64(40.5%)	109(29.5%)	137(27.0%)	317(29.9%)	10.543	0.01	0.01
Sepsis(n，%)	7(28.0%)	31(19.6%)	56(15.2%)	47(9.3%)	141(13.3%)	18.408	0.00	0.00

*BPD*=Bronchopulmonary dysplasia, *IVH*=Intraventricular hemorrhage, *PVL* = Periventricular leukomalacia, *NEC*=Necrotizing enterocolitis, *ROP* = Retinopathy of prematurity

## Discussion

There are many factors causing preterm delivery, [15, 16] such as maternal age, primary diabetes, hypertension, and other diseases. The results of this survey revealed an association between the older age of the mother and the lower birth weight of the newborn, which is consistent with previous reports, [17] It has been shown that the use of antenatal corticosteroids can improve neonatal outcomes, [18] especially for preterm infants with gestational age less than 34 weeks. However, only 54.5% of the infants in this study received prenatal hormonal therapy, which was similar to that in other low-income and middle-income countries [19]. This suggests that the cooperation between neonatology and obstetrics needs to be strengthened, and prenatal hormones should be recommended to improve the survival rate and prognosis of preterm infants in China.

Previous studies have also suggested a protective effect of noninvasive ventilation in preterm infants. CPAP is recommended as the preferred noninvasive ventilation mode after birth [20]. Early CPAP can avoid alveolar collapse and can reduce the chance of invasive ventilation [21]. From the previous study on preterm infants at 24 to 28 weeks of gestation, the proportion of using CPAP in the delivery room was 81.1%, [21] and in this study, CPAP was the most frequently used ventilation mode for VLBWIs, accounting for 65.2%. Previous studies have shown that preterm infant with gestational age < 28 weeks have a 50% probability of failure at first extubation, which may lead to higher mortality and morbidity [22]. The application of CPAP for support after extubation helps to reduce the re-intubation caused by extubation failure [23]. However, the proportion of preterm infants with birth weight less than 750 g who needed invasive ventilation reached 50% in this study.

In addition to noninvasive ventilation, caffeine administration also played major roles on promotion of neonatal morbidity. Caffeine can help VLBWIs wean earlier, reduce the incidence of BPD, and improve the prognosis of neurodevelopment [24–27]. In this study, 71.8% of VLBWIs were treated with caffeine. PS also has an important role in the respiratory tract management of VLBWIs [17]. The results of our study showed that 51.4% of VLBWIs received pulmonary surfactant (PS); the smaller birth weight was associated with a higher probability of using PS. Recent studies have shown that early use of budesonide nebulization can reduce the incidence of BPD in preterm infants [20, 28–30]. And in this study, the proportion of VLBWIs nebulization was only 19.4%. From our clinical practice, nebulization was usually administered before BPD developed in the infants. BPD was more likely to occur in infants with small birth weight, who used nebulization more frequently in our study Therefore, earlier atomization based on evaluation may be considered in the future. .

Adequate nutrition supply is the key for the survival of preterm infants. Proper vascular access is the key to parenteral nutrition but limited studies focus on the vascular choices in the NICU. UVC and PICC are the most commonly used vascular accesses for VLBWIs. In this study, UVC and PICC were used in 25.0 and 64.4% infants, respectively. Meanwhile, smaller birth weight was associated with the longer indwelling time of UVC and PICC, which was consistent with previous study [28]. In this study, the duration of parenteral nutrition in VLBWIs was 27.0 ± 19.5 days, the indwelling time of the feeding tube was 36.2 ± 21.0 days; smaller birth weight was associated with a longer need for parenteral nutrition and indwelling gastric tube.

In terms of enteral nutrition, breast milk is the first choice for VLBWIs, and it is related to the prognosis and survival rate [30, 31]. According to a survey in Jiangsu Province in 2018, the breast feeding rate of VLBWIs in the NICU of 29 hospitals was 37.2% [32]. In this study, the breast feeding rate of preterm infants with weight > 1250 g was 78.3%. Moreover, 66.8% of the preterm infants used their own mother’s milk, which was higher than that reported in other studies [33, 34]. The potential reason was the multidisciplinary team of breast milk management, including doctors, nurses, nutritionists, international breast milk consultants, and family members with breast feeding experience, which ensured the safety and successful implementation of breast feeding in NICU [35]. The corrected gestational age for full oral feeding was 35.3 ± 6.5 weeks, which was similar to the gestational age of full oral feeding in other studies [36]. However, the gestational age and weight of the study population included in other studies were relatively small, which suggested that if the gestational age and weight were the same, the time to achieve full oral feeding would be longer. At the same time, our results also revealed that the smaller birth weight was associated with the greater the corrected gestational age when reaching full oral feeding. Because birth weight is an important factor affecting the oral feeding process of preterm infants [37].

The management of body temperature is very important for preterm infants, in this study, hypothermia was observed in 19.3% of cases during hospitalization. We concluded that the smaller birth weight was associated with a higher incidence of hypothermia. Some researchers have suggested that bundles of golden hour temperature management including plastic bags usage, pre-warming the linens could significantly reduce the incidence of hypothermia in VLBWIs [38, 39]. In addition, hyperthermia was observed in 34.7% of total infants, and preterm infants with a birth weight of 750-1250 g had the highest probability of hyperthermia (42.2%). However, while we investigated whether hyperthermia occurred in the whole process of hospitalization, it was possible that there were numerous confounding factors.

EEG monitoring, brain or intestinal oxygen monitoring, and transcutaneous oxygen partial pressure monitoring are very important noninvasive monitoring methods in clinic. However, our results revealed that these monitoring methods were not frequently applied in VLBWIs, which also indicated that this kind of monitoring was not taken as routine monitoring in clinic. Infants with smaller birth weight and the more serious diseases were associated with the higher the proportion of such kind of monitoring.

The mortality rate of VLBWIs included in this study was 2.4%. Lower birth weight was associated with a higher mortality rate. Compared with other domestic studies, [1] the mortality in this study was relatively low. Because this study included only infants who died in hospital, and did not consider infants who died after discharge or due to withdrawal treatment. Apart from focus on the mortality of VLBWIs, it was necessary to pay attention to the incidence of various important short-term prognosis of the surviving infants. Compared with the domestic research results, [1] the incidence of BPD in this study was higher, but the incidence of sepsis was lower, which may be due to the role of collaborative quality improvement in the prevention of infection in preterm infants.

## Conclusion

This study described the care practices of VLBWIs in the NICU of the Yangtze River Delta region, thus providing baseline data for the care of VLBWIs in the future. Our results also showed that the care strategies of VLBWIs in the Yangtze River Delta region needed to be improved.

## Data Availability

The datasets used and/or analyzed during the current study were available from the corresponding author on reasonable request.
